# Where to Live Cheaply

**Published:** 1869-03

**Authors:** 


### WHERE TO LIVE CHEAPLY.

Go where you can purchase your clothing from the man who makes it; where you can buy your food from the man who raises it; where you can rent your house from the mechanic who built it; and so as to every other department of domestic expenditure; and thus get rid of the large army of middle men, of go-betweens, who produce nothing, do no work and yet manage to live on the work and money of the industrious and the economical. Between the farmer who raises a bullock or a pig and the man who eats it, there are three or four or more persons who manage to make profit, the farmer getting the least; and so it is with everything else needed for family consumption. Instead of renting our houses from the persons who own them we must do it through an agent, who must rent an office down town, at an expense of one or two thousand dollars a year; must pay as much for clerk hire, to say nothing of his own family residence, up town, to which must be added family expenses, amounting to five or ten thousand dollars a year, which must be paid by those who rent; nominally the owner pays it, but knowing what the custom is, he puts that much more on the price of his house before he puts it into the market.

That class of persons who have a fixed income, and are out of business, especially if they have children to educate, will find it a money-saving operation to go abroad.

GERMANY

Is the Mecca of all who wish to live comfortably on comparatively small means. As we have recently been over the ground, we will give some items which will interest some of our readers.

In Bonn, Manheim, Heidelberg and other places on or near the Rhine, there are numerous schools, colleges and universities, numbering a thousand students, where living is comparatively cheap, and the opportunities of education very great.

A fine double house, three stories, with large gardens on each side, and lawn, extending to the banks of the river, completely furnished, was pointed out by a citizen as renting for five hundred gold dollars a year; superior cooks at forty dollars a year, and housemaids for about twenty-five.

In Manheim there is a boarding-school for young ladies, patronized by the nobility and under the supervision of the Duchess of the country and her generous husband; by their joint liberalities, the inmates pay only for board, washing and some other items; the entire cost being only two hundred and forty gold dollars a year. At

HEIDELBERG,

Ten miles from the Rhine, by rail, which has so much about it that is historic, and quaint and beautiful and grand; the abode of royalty for centuries; where many a time and oft contending armies have made its streets all black and desolate with the havoc of war and fire and blood; beautiful and classic Heidelberg, with its pleasing memories, has been repeatedly burned to ashes, razed to its very foundations, and again and again bombarded, but is still grand in its ruins, and in its memories; here fully competent instructors in music, painting, drawing, sketching, modern languages, in short, every branch is represented, for about forty cents an hour, and sometimes lower. A family of four, with parlor, dining-room and four chambers, very pleasantly furnished; their meals all brought, at stated times, fresh and hot, with servants to remove everything promptly, pay about twenty dollars a week in currency; living as retired as if in their own private dwellings, the houses being so constructed that each floor is distinct from the other portions of the building, and no one enters your domain except yourself.

While there, for a short time, we had occasion to order a a pair of gaiter-boots; they were made to measure, after the New York fashion, by a man who had made shoes in New York twelve years, for two dollars and half in gold a pair; other things are in proportion. We found quite a large number of American and English families there; the Episcopal church was filled every Sabbath day, the rector being an English gentleman of education and ability and a good man; the vine-clad hills tower above the town from one to two thousand feet in luxuriant verdure, while the peaceful Neckar glides away into the plain below in its silvery brightness, gladdening the eye and crowding the mind with peaceful scenery; that is, writing travelers would rattle on in this style, but what attracted our attention was the comparative immorality of New York with this now peaceful, quiet, old town, where they have no fences in its suburbs, the fields being separated by a simple path, and their market garden spots are divided from the great highway by a patch of grass a few inches wide; while in New York neither wooden fences forty feet high, nor stone walls a yard thick, nor iron bars, nor cavalcades of policemen, nor any other human safe-guard can prevent depredations on the property and premises of peaceful, honest citizens. It really seems that good, honest, well-meaning people of moderate means would be gainers, morally, socially, civilly and pecuniarily by emigrating, in a body, to honest, plodding, peaceful Germany, and in the blessed feeling of abiding security and low prices, spend the remnant of their days, while their children are prosecuting their studies in quietude, playfulness and peace.

LIVING IN DRESDEN,

The city of the court, is more expensive, but to many more enjoyable, as it has fifty thousand inhabitants; has extensive libraries and picture galleries; where the opera and other public amusements attract multitudes of strangers to the place; it is probably the most enjoyable city in Europe; it would at least seem so from the large number of American and English families, who reside there permanently.

The facilities for the education of both sexes are very great in Dresden. The houses are adapted to the acommodation of a number of families, each family occupying a "floor," as it is called there, we term them stories, but so arranged that each floor is a complete house in itself, with parlor, dining-room, chambers, kitchen, closets, etc. One of these stories, completely furnished with greater or less elegance, can be had for thirty-five to one hundred dollars a month. A first floor of one of the finest residences in the city, containing seventeen rooms, rents for eleven hundred dollars a year, in our paper money; the third story, of eight rooms, nicely furnished, brings three hundred paper dollars a year. Many American and English families are living in first and second stories, with rooms, linen, kitchen utensils and furniture for five hundred paper dollars a year. Servants' wages are low, cooks for three to six dollars a month, not a week, but a month; chickens and eggs are very low. To live in Dresden in about the same style we would live in New York, costs from one half to two-thirds less than it does here. The charges for

### SCHOOLS AND TEACHERS

Are very reasonable. Piano lessons are given there for one and two dollars, which instructors in Boston and New York, of no greater ability, charge five dollars an hour for. One of the best female teachers in Dresden charges fifty paper cents an hour, and the same for the best German teachers.

### CLOTHING.

Suits and overcoats, which cost here from seventy to eighty dollars, cost less than thirty-five there, while our fifteen and twenty-dollar laced boots are had there for five or six; the best shirts are two-fifty each; the best German kid gloves cost one dollar; a drive in a public vehicle costs twelve cents; an opera box costs one dollar and twenty-five cents, and concert tickets from a dime to a dollar.

### SEA-VOYAGE.

There are a good many persons in the United States who have no money to throw away, having earned it honestly in legitimate business and after many years of economy and self-denial find themselves with little more than they have use for and would like to make an economical trip across the ocean. A man can go from New York to Heidelberg for a hundred and forty paper dollars or less; they take passage in the "Anchor Line" of steamers to Glasgow, ninety dollars; to Edinburgh, two; to Rotterdam, at the mouth of the Rhine, ten; up the whole length of the Rhine passing Cologne, Bingen, the mouth of the "Blue Moselle," and various other places of great interest, to Manheim, being three nights on the way, when the boats lay by, ten dollars; from Manheim to Heidelberg, by rail, ten miles, for which last and extra or unexpected expenses also ten dollars more. If a quicker and more direct passage is desired, take a New York and German steamer to Hamburgh or Bremen, one hundred and twenty dollars in gold, thence to Dresden or Heidelberg direct by rail for about fifteen dollars in gold. If persons wish to go by London or Liverpool, the cost is about a hundred dollars in gold; from London to Heidelberg is about fifteen dollars in gold. But this is enough about foreign travel in one article, at another time other things may be said. But there was one great comfort in Scotland, London and Germany, that of being able to go to bed with a feeling of perfect security, without the necessity of locking your chamber door, then bolting it, then putting your bedstead against it, and after all having a loaded revolver which will shoot yourself and half a dozen others, in the time you could say "Jack Robinson," as is the case in New York.

